# Clinicopathological Profile of Intraosseous Adenoid Cystic Carcinoma of the Jaws: A Systematic Review

**DOI:** 10.1111/jop.70063

**Published:** 2025-09-23

**Authors:** Luccas Lavareze, João Figueira Scarini, Reydson Alcides de Lima‐Souza, Talita de Carvalho Kimura, Rogério de Oliveira Gondak, Erika Said Abu Egal, Albina Altemani, Fernanda Viviane Mariano

**Affiliations:** ^1^ Department of Pathology, School of Medical Sciences University of Campinas (UNICAMP) São Paulo Brazil; ^2^ Department of Oral Diagnosis, Piracicaba Dental School University of Campinas (UNICAMP) Piracicaba Brazil; ^3^ Department of Pathology Federal University of Santa Catarina Florianopolis Brazil; ^4^ Department of Pathology, School of Medicine University of Utah (UU) Salt Lake City Utah USA

**Keywords:** adenoid cystic carcinoma, jaws tumors, primary intraosseous adenoid cystic carcinoma, salivary gland cancer, systematic review

## Abstract

**Objectives:**

We aimed to evaluate the clinicopathological features, survival rate, and potential prognostic markers of the jaws' primary intraosseous adenoid cystic carcinoma (PIACC).

**Materials and Methods:**

MEDLINE/PubMed, Scopus, and Embase searches were performed with the keywords “adenoid cystic carcinoma,” and “jaw,” or “maxilla,” or “mandible.” We included articles that evaluated cases diagnosed as PIACC in jaws. Studies with insufficient demographic data, inconclusive histopathologic diagnosis, and appropriate follow‐up were excluded. The Joanna Briggs Institute tool was used to assess the risk of bias.

**Results:**

Fifty‐five PIACC comprising 27 studies met the inclusion criteria. The mean age of the patients was 56.4 ± 19.6 years with no sex predilection. PIACC showed a strong predilection for the mandible (69.1%), mainly in the posterior segment (40%). The patients presented symptoms in 87.3% of cases. Radiographically, PIACC presented as an ill‐defined radiolucent lesion (40%). Most cases showed a cribriform pattern (32.7%). PIACC with a solid growth pattern presented a lower disease‐free survival (DFS) (*p =* 0.040). The 2‐ and 5‐year overall survival rates were 57.9% and 53.8%, respectively. Distant metastases were seen in 3.6% of the patients and were related to a lower DFS (*p =* 0.043).

**Conclusion:**

PIACC is a rare neoplasm of the jaws with an incidence in the fifth and sixth decades of life and no sex predilection. The posterior mandible was affected in most cases. Solid growth patterns and distant metastases are prognostic factors for a lower DFS.

## 1 Introduction

Malignant central salivary gland tumors are a rare group of lesions that represent less than 0.4% of all salivary gland cancers. These tumors usually show the same morphology as those found in salivary gland tumors [1, 2]. The etiology of malignant central salivary gland tumors is unknown, but hypotheses such as the ectopic salivary gland and the origin of the odontogenic epithelium have been reported [3, 4].

Adenoid cystic carcinoma (ACC) is the second most common salivary gland cancer and is known for its slow growth and infiltration behaviour [5]. Despite being more incident in parotid glands, ACC has already been described in other glandular tissues and the jaws [1, 2, 5]. Primary intraosseous adenoid cystic carcinoma (PIACC) is characterized by intraosseous tumor growth with similar ACC gland features, without a history of metastatic tumor or extraosseous origin [2, 6]. Despite being described in the literature since the 1950s [7], PIACC is still a poorly characterized entity with unknown clinical behavior. In this systematic review, we aimed to evaluate the clinicopathological features, survival rate, and potential prognostic markers of PIACC of the jaws. The review question was “What is the clinicopathological, prognostic, and survival profile of patients with intraosseous adenoid cystic carcinoma of the jaws?” No similar systematic review has been published.

## 2 Methodology

### 2.1 Protocol and Registration

This systematic review was based on the Preferred Reporting Items for Systematic Reviews and Meta‐Analyses (PRISMA) 2020 checklist [8] and was previously registered in the International Prospective Register of Systematic Reviews (PROSPERO) database (CRD42022363519).

### 2.2 Eligibility Criteria

We included articles that evaluated cases diagnosed by histopathological examination of PIACC located in the maxilla or mandible. The studies should present clinicopathological and/or radiographic evidence that the tumor originated within the jaws. Cohort studies, cross‐sectional studies, case–control studies, clinical trials, case reports, and case series, published in English, Portuguese, or Spanish, were included. In case series and crossover studies, only patients who met the eligibility criteria were selected. Studies with insufficient demographic data, inconclusive histopathologic diagnosis, appropriate follow‐up, tumors with a suspected extraosseous origin, conference abstracts, and letters to the editor were excluded.

### 2.3 Information Sources and Search Strategy

An electronic search was conducted on November 17, 2022, and updated on November 7, 2023, on the MEDLINE by PubMed, Scopus, and EMBASE data platforms without any period restriction. The search strategies were as follows: [(“adenoid cystic carcinoma”) AND (“jaw” OR “maxilla” OR “mandible” OR “mandibular” OR “jaws” OR “intraosseous” OR “central”)] in PubMed/MEDLINE and EMBASE, and TITLE‐ABS‐KEY [(“adenoid cystic carcinoma”) AND (“jaw” OR “maxilla” OR “mandible” OR “mandibular” OR “jaws” OR “intraosseous” OR “central”)] in Scopus. A manual search in Google Scholar and the reference list of the included articles was performed for additional studies addition. The complete search strategy is described in Table S1.

### 2.4 Study Selection Process and Data Collection

Rayyan QCRI12 was used to screen the articles and remove duplicates. Two independent authors performed the selection of articles that met the eligibility criteria by the titles and abstracts. After that, a complete analysis of the studies was performed by applying the eligibility criteria and recording the reasons for exclusion (Table S2). Disagreements were first resolved by discussion among the authors and cases with no agreement were consulted by a third author.

The extracted data were added to a Microsoft Excel table by one author and reviewed by a second author. For each selected study, the following information was recorded (when available): author(s), year of publication, country, type of study, total number of eligible patients, gender and age, tumor location, clinical presentation and symptoms, duration of manifestations, local and distant metastases at diagnosis, tumor growth pattern, treatment, follow‐up time, outcome, and patient status at last follow‐up.

### 2.5 Risk of Bias in Individual Studies

The Critical Appraisal tool from the Joanna Briggs Institute [9] was used to assess the risk of bias in the selected studies. Two independent authors classified the articles as having a “high,” “moderate,” or “low” risk of bias according to the tool's criteria. Studies with complete clinicopathological data, description of the outcome and follow‐up, clear inclusion and exclusion criteria, and valid statistical analysis, when applicable, were considered to have a low risk of bias. Disagreements were first resolved by discussion among the authors, and cases with no agreement were consulted by a third author.

### 2.6 Statistical Analysis

The qualitative and quantitative data were presented descriptively using percentage, mean, and standard deviation. Only patients with appropriate follow‐up and outcome descriptions were selected for statistical analysis of disease‐free survival (DFS) and overall survival (OS). Data were analyzed using the Statistical Package for Social Sciences (SPSS, IBM Corporation, Armonk, NY) software, version 23.0. The Kaplan–Meier curve and log‐rank tests estimated the DFS. The correlation of clinicopathological characteristics with patient status was performed by the Pearson Chi‐square test, and a *p* < 0.05 was considered statistically significant. The univariate Cox proportional hazard regression model was employed to identify potential prognostic factors. All statistically significant variables were applied to the multivariate Cox proportional hazards model.

## 3 Results

### 3.1 Selection and Characteristics of the Studies

**FIGURE 1:**
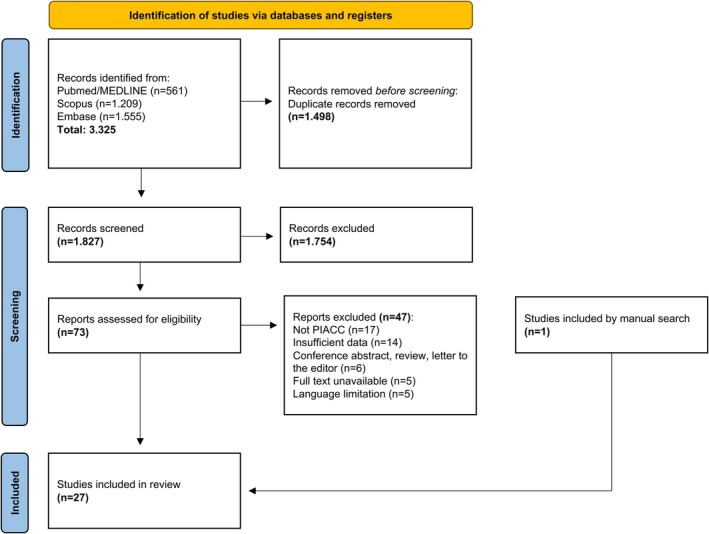
Flowchart of the selected studies adapted from PRISMA 2020 [8].

The flowchart illustrating the selection of articles is summarized in Figure 1. The search strategy resulted in 3325 articles from the period 1955 to 2022. After removing duplicates, 1827 articles remained for screening. After reading the title and abstract, 74 articles were selected for full reading, with 27 selected for data extraction. One article was selected in a manual search on Google Academic. Finally, 27 articles (55 patients) were included and distributed in 20 case reports and seven case series (Table S3). Most of the studies were excluded due to a lack of evidence regarding the intraosseous nature of the tumor or incomplete follow‐up (Table S2). The total number of patients per study ranged from 1 to 16. The selected articles originated from 13 countries: China (five), Japan (four), USA (four), India (three), Brazil (two), UK (two), Sudan (one), Germany (one), Italy (one), Finland (one), Spain (one), Mexico (one), South Africa (one). No other study was included in the 2023 update.

### 3.2 Risk of Bias

The risk of bias was applied to 20 case reports and seven case series, resulting in 20 (74.1%) publications with a low risk of bias and seven (25.9%) with a moderate risk of bias. Most of the studies that presented a moderate risk of bias had limitations in methodology and statistical analysis description, aligned with an incomplete clinicopathological description. The description of the individual risk of bias for each study is in Figure S1.

### 3.3 Clinical and Demographic Characteristics

**FIGURE 2:**
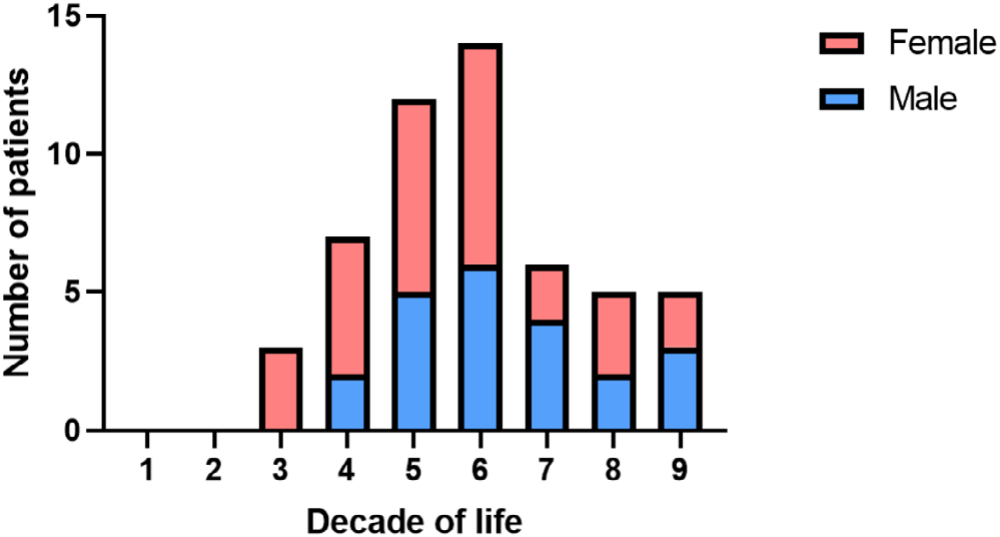
PIACC distribution in the decade of life and sex.

The clinicopathological characteristics of the 55 patients included in the review are summarized in Table 1. Detailed information on each study is in Table S3. There were 30 women (54.5%) and 25 men (45.4%). The male‐to‐female ratio was 1:1.2. The patients' mean age at diagnosis was 54.2 ± 15.3 years (range 24–82 years), with a peak in the fifth and sixth decades of life (Figure 2).

**TABLE 1:** Clinicopathological characteristics of the PIACC patients.

Variables	Value (%)
Sex	
Male	25 (45.4)
Female	30 (54.5)
Age	
Age ≤ 40	10 (18.2)
Age > 40	45 (81.8)
Site	
Maxilla	17 (30.9)
Mandible	38 (69.1)
Presence of symptoms	
No	3 (5.5)
Yes	48 (87.3)
NA	4 (7.3)
Clinical presentation	
Intraoral swelling	15 (27.3)
Intra‐ and extraoral swelling	5 (9.1)
Other	5 (9.1)
NA	30 (54.5)
Size	
≤ 2 cm	2 (3.6)
> 2 cm but ≤ 4 cm	14 (25.4)
> 4 cm	13 (26.6)
NA	26 (47.2)
Palpable lymph nodes	
Yes	10 (18.2)
No	26 (47.3)
NA	19 (34.5)
Long distance metastasis	
Yes	4 (7.4)
No	9 (16.6)
NA	42 (76.4)
Radiographic finding	
Mixed area	2 (3.6)
Well‐defined radiolucent area	7 (12.7)
Poor defined radiolucent	22 (40)
NA	24 (43.6)
Histological subtype	
Tubular	2 (3.6)
Solid	15 (27.3)
Cribriform	18 (32.7)
Mixed	6 (10.9)
NA	14 (25.5)
Invasion	
Yes	32 (58.2)
No	4 (7.3)
NA	19 (34.5)
Perineural invasion	
Yes	14 (25.5)
No	7 (12.7)
NA	34 (61.8)
Primary treatment	
Surgery alone	10 (18.2)
Combined treatment	38 (69.1)
Other^a^	7 (12.7)
Patient status	
Dead	12 (21.8)
Alive	43 (78.2)

Abbreviation: NA, not available.

^a^ Four cases of radiation therapy, two cases of chemotherapy, and one case of no treatment.

PIACC showed a strong predilection for the mandible (69.1%), mainly in the posterior segment (40%). The maxilla was affected in 30.9% of cases. Most PIACC presented as intraoral swelling (27.3%), usually with no ulcerations. Other presentations were extraoral and intraoral swelling and tooth mobility. The patients presented symptoms in 87.3% of cases, with pain and paresthesia being the most reported complaints. Other symptoms associated with PIACC were swelling, toothache, tooth loss, and epistaxis. The mean duration of symptoms was 11.5 ± 11.5 months (1–48 months). Lymph nodes enlargement were reported in 18.2% of the patients, while distant metastasis was reported in 7.4% of the patients at the time of diagnosis. Lungs were the most affected site of metastasis in 75% of patients.

### 3.4 Radiographic Characteristics

PIACC is presented in most cases as a radiolucent destructive lesion with ill‐defined limits (40%). Other radiographic presentations were well‐defined radiolucent lesions (12.7%) and mixed lesions (3.6%). “Floating teeth” were reported in two cases [10]. There was no association with impacted teeth. Most of the lesions were larger than 2 cm (52%). Three multifocal PIACC were described in the mandible [11, 12, 13]. Benign conditions were the initial hypothesis in 10 patients, malignant neoplasms in seven patients, and periapical infection in five cases.

### 3.5 Histopathological and Molecular Characteristics

**FIGURE 3:**
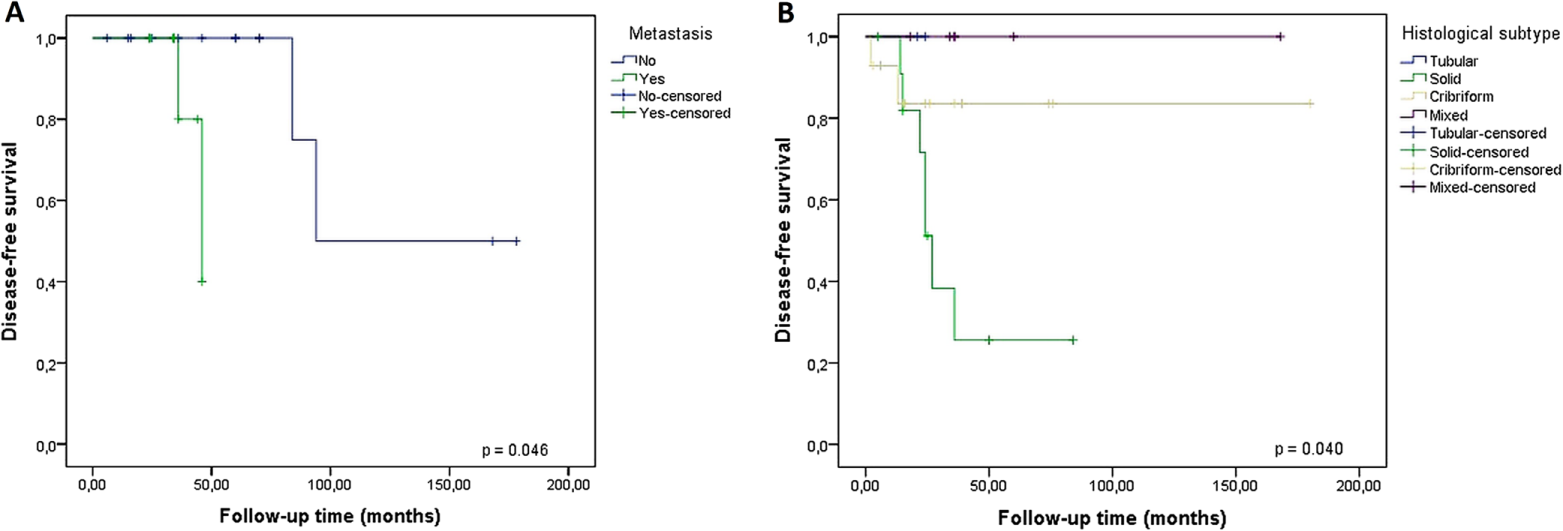
Kaplan–Meyer disease‐free survival curve using univariate Log‐Rank analysis for (A) metastasis (*p* = 0.046) and (B) histological subtype (*p* = 0.040).

Histologically, PIACC presented as an invasive lesion in adjacent tissues (58.2%), generally presenting perineural invasion (66.6% of studies that presented the description of perineural invasion). The tumor showed small darkly stained cubic, ovoid, or polyhedral cells with scant cytoplasm and ill‐defined cell borders. The nuclei were small, round, or ovoid, showing clumped or dispersed chromatin [10, 13, 14, 15, 16, 17, 18, 19]. The nucleoli varied from indistinct to evident. These cells were surrounded by evident basal lamina deposition and hyaline stroma. In some PIACC, the cells were organized in “pseudo‐cystic” spaces filled with hyaline or mucoid material, giving the tumor the cribriform or tubular pattern [10, 13, 14, 15, 16, 17, 18, 19, 20]. The predominance of the cribriform pattern was found in 32.7% of cases, followed by the solid pattern in 27.3%. Mixed and the tubular growth pattern was described in 10.9% and 3.6% of tumors, respectively. In 25.5% of the cases, the description of the histological pattern was absent. In univariate analysis, PIACC with a solid growth pattern presented a lower DFS (*p =* 0.040) (Figure 3 and Table 2). However, this variable was not statistically significant in the multivariate analysis (*p =* 0.090) (Table 3). The luminal epithelial cells showed positive immunostaining for AE1‐AE3 [15, 21], CAM 5.2 [21], CK7 [15, 22], CK17 [15], CK19 [15, 22], CD117 [22], β‐catenin [15], E‐cadherin [15], BCL‐2 [15] while the myoepithelial cells were positive for calponin [22], S100 [15, 16], smooth muscle alpha‐actin (SMA) [15], CD43 [15], epithelial membrane antigen (EMA) [15], and p63 [15, 22]. The Ki‐67 was 3% [15]. Fluorescent in situ hybridization (FISH) analyses showed *MYB* abnormalities (break of *MYB* gene), amplification or loss of 5′ part in all PIACC (four tumors) presented in a case series [22].

**TABLE 2:** Log‐rank univariate analysis of the clinicopathological characteristics of 55 cases of PIACC.

Clinicopathological variables	Log‐rank univariate analysis			
	5‐year disease‐free survival (%)	Estimate (95% CI)	Chi‐square	*p*
Sex				
Male	65.2	109.5 (67.5–151.6)	1.198	0.274
Female	81.0	125.2 (77.0–173.3)		
Age				
Age ≤ 40	83.3	152.3 (102.8–201.8)	0.163	0.686
Age > 40	71.1	112.0 (73.0–151.0)		
Site				
Maxilla	66.7	114.0 (67.7–160.2)	0.392	0.531
Mandible	75.9	113.3 (65.4–161.2)		
Presence of symptoms				
No	100.0	63.0 (42.0–83.9)	0.274	0.601
Yes	74.4	120.2 (79.9–160.5)		
Clinical presentation				
Intraoral swelling	72.7	97.4 (43.7–151.1)	0.067	0.796
Intra‐ and extraoral swelling	66.7	45.5 (20.8–70.1)		
Size				
≤ 2 cm	100.0	42.0 (19.5–141.0)	2.143	0.343
> 2 cm but ≤ 4 cm	100.0	36.0 (24.0–50.0)		
> 4 cm	80.0	27.5 (23.2–59.0)		
Positive lymph nodes				
Yes	88.9	157.7 (117.2–198.1)	0.879	0.349
No	75.0	95.85 (47.0–144.7)		
Long distance metastasis				
Yes	66.7	36.0 (0–72.0)	4.000	0.046
No	85.7	126.0 (67.7–184.2)		
Radiographic finding				
Mixed area	100.0	98.7 (61.5–135.8)	5.144	0.076
Well‐defined radiolucent area	100.0	117.6 (72.3–162.9)		
Poor defined radiolucent	57.9	61.3 (49.1–73.5)		
Histological subtype				
Tubular	100.0	134.5 (40.9–228.0)	8.317	0.040
Solid	41.7	57.0 (38.1–75.9)		
Cribriform	85.7	140.1 (98.3–181.8)		
Mixed	100.0	117.5 (47.5–187.4)		
Primary treatment				
Surgery alone	80.0	152.4 (104.3–200.5)	2.535	0.282
Combined treatment	75.9	95.4 (45.5–70.7)		

Abbreviation: CI, confidence interval.

**TABLE 3:** Multivariate analysis for disease‐free survival of PIACC patients.

Variables	HR multivariate (95% CI)	*p*
Long distance metastasis		
Yes	1.70 (0.17–2.88)	0.073
No	1.0 (Ref.)	
Histological subtype		
Tubular	1.07 (0.06–17.1)	0.090
Solid	4.46 (0.57–34.6)	
Cribriform	1.38 (0.14–13.3)	
Mixed	1.0 (Ref.)	

Abbreviations: CI, confidence interval; HR, hazard ratio.

### 3.6 Treatment and Prognosis

The primary therapy modalities were varied across the studies. Most cases were treated with a combination of therapies (69.1%), with surgery and neck dissection with or without radiation therapy (RT) being the most used (28.9%). Chemotherapy (CT) was used as adjuvant therapy in five cases. A single treatment modality was used in 29% of cases, and surgery alone was the most reported (62.5%), followed by RT (31.2%) and CT (6.2%). One patient refused treatment [22].

The mean follow‐up was 44 ± 42.9 months (2–192 months). Of the 55 cases, only 38 had follow‐ups longer than 24 months and 13 longer than 60 months. Across these patients, the 2‐year OS was 57.9% while the 5‐year OS was 53.8%. Local recurrence was seen in 12.7%, while local and distant metastases were seen in 3.6% and 12.7% of cases, respectively. Lung and bone were the most affected metastasis sites. In univariate analysis, patients with distant metastases had a lower DFS (*p =* 0.043) (Figure 3 and Table 2). However, this variable was not statistically significant in the multivariate analysis (*p =* 0.073) (Table 3). In cases where the patient had recurrence or metastasis, the mean time between the end of the initial treatment and the outcome was 39.2 months (1–144 months).

Overall, during the follow‐up period, 32 patients had no evidence of disease (58.2%), 11 patients were alive in treatment (20%), and one died from other causes (1.8%). Eleven patients died from the disease (20%), and five showed recurrence or metastasis.

## 4 Discussion

Intraosseous malignant salivary gland tumors of the jaw bones are a rare group of tumors, representing less than 0.4% of all salivary gland cancers. Central mucoepidermoid carcinoma is the most commonly reported; however, several other histological subtypes have been described in the literature [1, 2]. Studies indicate that PIACC is the second most frequent salivary gland cancer in the jaws [2, 6], similar to the ACC incidence in salivary glands [23]. In this review, we highlighted features of PIACC that may exhibit similarities with the salivary gland ACC (SACC).

ACC is a malignant tumor that can occur in several glandular tissues of the body, such as mammary and lacrimal glands. The site of incidence of the tumor is usually related to its clinical behavior and prognosis [5]. The salivary gland is the most affected site by ACC, and despite its slow growth, SACC presents a potential for invasion, tissue destruction, and metastasis [5, 24]. SACC presents a slight predilection for females, with a predominance in the sixth and seventh decades of life [25, 26] similar to our results. Generally, SACC has a higher incidence in major salivary glands, especially the parotid gland [25]. In this review, PIACC showed a marked predilection for the mandible.

The interesting features of intraosseous salivary gland cancers origin are related to their etiology and histogenesis. While ACC has an origin in intercalated duct cells in glandular tissues, these structures are not components of the bone tissue [5, 24, 27]. The absence of histological evidence of salivary glandular tissue in the bone marrow led to the hypothesis that intraosseous salivary gland tumors could originate from ectopic salivary glandular tissue. During embryogenesis, the sublingual gland or the minor salivary glands of the retromolar region tissue are trapped within the cortical bone [3, 4]. Nevertheless, the absence of findings of glandular tissue in intraosseous biopsies or adjacent to intraosseous glandular tumors ruled out this hypothesis [3, 4]. Moreover, this hypothesis would not consider tumors that are not near the retromolar and mandibular body regions. A further study conducted by Bouquot et al. found a 0.3% incidence of ectopic salivary glandular tissue in 5034 intraosseous biopsies [4]. Besides the low incidence of ectopic glandular tissue, intraosseous salivary tumors are rare and in limited cases may originate from these tissues.

Currently, the most accepted hypothesis is that salivary glandular cancer in the jaws arises from pluripotential stem cells present in the periodontal ligament or dental lamina [3]. This theory is reinforced by the presence of other salivary gland cells in the epithelia of odontogenic lesions, including mucous or metaplastic calyceal cells. These cells may be seen in lesions such as periapical cysts, glandular odontogenic cysts [3, 28], and also the malignant transformation of odontogenic lesions to salivary gland cancer [3, 29]. In this review, no PIACC was found associated with impacted teeth, reducing the likelihood of representing an ACC arising from a prior benign odontogenic lesion. Although PIACCs resembling periapical lesions were relatively frequent [17, 20, 21, 30, 31, 32], no study documented the presence of residual lesions in the histological examination. This finding reinforces the concept that the tumors were primary.

Regarding clinical presentation, ACC is typically associated with painful symptoms or paresthesia due to its potential for perineural invasion [33]. These characteristics can also be observed in PIACC, where symptoms and perineural invasion were reported in 87.3% and 66.6% of the cases, respectively. In our review, five patients were initially treated for odontogenic infection due to painful symptoms and radiographic findings [17, 20, 21, 30, 31, 32]. Clinicians should be vigilant for other clinical symptoms of infection such as fever, edema, and erythema to avoid delays in diagnosis and treatment of neoplastic lesions. Regarding perineural invasion, in ACC this finding is an important predictor of a poorer prognosis, often associated with metastasis and reduced survival rates [26]; however, in our cases, there is no association between perineural invasion and a lower DFS.

SACC may be associated with pain or paresthesia due to perineural invasion, even in early stages [33], being a predictor for a poorer prognosis concerning metastasis and reduced survival [26]. According to the World Health Organization (WHO‐2017), the perineural invasion is a hallmark of SACC [34] but its mechanism is not fully understood. Studies showed that the perineural invasion may occur due to the low resistance path of nerves for metastasis or signaling factors that enhance malignant cell affinity for nerves [33, 35]. Besides the method, the perineural invasion seems to be also present in PIACC. In our review, 66.6% of patients exhibited perineural invasion when the data was available in the manuscript. The painful symptoms and a radiographic destructive image near the periapical region resulted in five PIACC being initially treated as odontogenic infections. Clinicians should be cautious in the management of osteolytic lesions in the jaws that do not respond to conventional treatment for odontogenic infections or are not associated with other inflammatory symptoms, such as heat, redness, and edema.

With regards to the histopathological presentation, PIACC shares no morphological distinctions from its glandular counterparts. ACC is composed of two types of primary cells: ductal cells with eosinophilic cytoplasm and uniform round nuclei, and myoepithelial cells with clear cytoplasm and hyperchromatic angular nuclei [34]. To diagnose ACC, it is required to identify both pseudocysts and true glandular lumina [34]. PIACC shows similar features such as small, darkly stained cells with ill‐defined borders and pseudocyst formation [10, 13, 14, 15, 16, 17, 18, 19, 20]. However, the selected articles poorly described the cytological difference between the ductal and myoepithelial cells. The tumoral cells may be organized in solid, cribriform, and ductal growth patterns [34]. Solid tumors are the least differentiated and most aggressive clinically and are usually associated with a poorer prognosis [5, 36, 37, 38]. Our results also showed that solid PIACC exhibited a poorer prognosis compared to cribriform and ductal patterns. Recently, mutations in the *NOTCH* pathway were identified in solid SACC and were associated with the loss of myoepithelial differentiation and a worse prognosis [36, 39]. In certain salivary gland tumors, such as carcinoma ex pleomorphic adenoma, myoepithelial cells seem to function as tumor suppressors in their early stages [40] and may also play a role in the ACC progression. Besides the morphology, both *MYB* rearrangement and MYB protein expression were observed in PIACC, further supporting a common molecular origin [22, 41]. Despite the absence of an exclusive histological, immunohistochemical, and molecular marker to PIACC, its similarities with the glandular ACC may allow the diagnostic markers to be shared by these tumors.

ACC is a type of tumor that is recognized for its ability to invade the surrounding tissues [34]. In cases where the tumor invades tissues beyond what is identified clinically and radiographically, the complete surgical excision may not be achieved. In a study by Amit et al., 59% of patients with SACC in its early stages demonstrated surgical margins compromised by the tumor. These patients demonstrated a 5‐year OS that was statistically lower than patients with negative margins [42]. In addition to the surgical challenge, tumors with positive margins are more likely to require adjuvant treatment and have locoregional recurrence [42]. In this review, PIACC demonstrated invasion and locoregional recurrence in 58.2% and 12.7% of the cases, respectively, showing that this tumor may be a management challenge.

While ACC typically follows a favorable clinical course, metastatic tumors pose a challenge [25] due to chemoresistance and the limitation of the therapeutic options [24, 43]. Nevertheless, in our review, CT was still employed in a significant portion of patients. Currently, radical surgery with or without RT remains the primary treatment for ACC, while metastatic tumors may necessitate metastasectomy [24]. Even when tyrosine kinase inhibitors and immune checkpoint inhibitors are a treatment option in some head and neck cancers [44], in ACC this modality of treatment still presented limited results and is often not considered [24, 45]. Despite slow tumor progression, SACC survival rates are low with only 45.1% of the patients surviving after 10 years. This percentage decreases even further in the 15‐year follow‐up [25]. The lack of treatment options for advanced ACC, particularly in the case of metastatic tumors, may contribute to a lower DFS rate for both ACC and PIACC. Especially in PIACC, the OS was similar to SACC survival rates, predicting an uncertain clinical behavior of this tumor.

As PIACC is a rare neoplasm, this systematic review relied mainly on case reports and case series. The primary limitation of this study lies in the heterogeneity of clinical and histopathological descriptions of the lesions, which may result in tumor characteristics going unreported. The lack of detailed clinical information may represent a limitation in the interpretation of maxillary PIACC. Since ACC may originate from the glandular structures of the maxillary sinus mucosa, a tumor in this region may be misinterpreted as intraosseous. Furthermore, many cases had a limited follow‐up, which constrains the availability of statistical data concerning survival and prognostic factors. It is essential to recognize that in the case of rare diseases, comprehensive descriptions are important for researchers and clinicians. Given the lack of comprehensive information on PIACC, reviews like this one offer an overview for clinicians and pathologists to better understand the disease, enhancing its diagnosis and treatment.

PIAAC still lacks more in‐depth information about diagnostic and prognostic markers. Further studies focused on the *MYB* alterations may provide information about the molecular origin of the tumor and a possible marker for diagnosis. Furthermore, comparative studies between PIACC and SACC may assist in the detection of specific markers for PIACC, such as tumor size, histological subtype, and perineural invasion, aiding pathologists and clinicians in tumor management.

## 5 Conclusion

PIACC is a rare neoplasm of the jaws, with no sex predilection and an incidence in the fifth and sixth decades of life (mean age 54.2 years). The posterior mandible was affected in most cases. PIACC presented as intraoral swelling usually related to pain or paresthesia. Radiographically, the tumor exhibits a destructive ill‐defined radiolucency. In histopathological analysis, PIACC showed potential for tissue and perineural invasion. Solid tumors and distant metastasis are potential prognostic factors that may lead to a lower DFS.

## Author Contributions

Luccas Lavareze, João Figueira Scarini, Reydson Alcides de Lima‐Souza, and Fernanda Viviane Mariano contributed to the conception, design, data acquisition, interpretation of data, writing, and critical revision of the manuscript. Talita de Carvalho Kimura contributed to the design and critical revision of the manuscript. Erika Said Abu Egal, Rogério de Oliveira Gondak, and Albina Altemani contributed to the interpretation of data and critical revision of the manuscript. All authors gave their final approval and agreed to be responsible for all aspects of the work.

## Conflicts of Interest

The authors declare no conflicts of interest.

## Supporting information


**Table S1:** Search strategy.
**Table S2:** Excluded articles.
**Table S3:** Included articles.
**Figure S1:** Risk of bias (Joanna Briggs Institute).

## Data Availability

The data that support the findings of this study are available from the corresponding author upon reasonable request.
