# Supplementary material for: Clinicopathological Profile of Intraosseous Adenoid Cystic Carcinoma of the Jaws: A Systematic Review
Source: J Oral Pathol Med. 2025 Sep 23;55(1):32–41. doi: 10.1111/jop.70063 (PMC12774579; doi:10.1111/jop.70063)
Supplement: Supplementary file 1 — Table S1: Search strategy. Table S2: Excluded articles. Table S3: Included articles. Figure S1: Risk of bias (Joanna Briggs Institute). [file JOP-55-32-s001.docx]

**Table S1.** Search strategy

| **Database** | **Search strategy**  (Search date: November 17, 2022) | **Results** |
| --- | --- | --- |
| **PubMed** | ("adenoid cystic carcinoma") AND ("jaw" OR "maxilla" OR "mandible" OR “mandibular” OR "jaws" OR "intraosseous" OR "central") | **561** |
| **Scopus** | TITLE-ABS-KEY (("adenoid cystic carcinoma") AND ("jaw" OR "maxilla" OR "mandible" OR “mandibular” OR "jaws" OR "intraosseous" OR "central")) | **1,209** |
| **Embase** | ('adenoid cystic carcinoma'/exp OR 'adenoid cystic carcinoma') AND ('jaw'/exp OR 'jaw' OR 'maxilla'/exp OR 'maxilla' OR 'mandible'/exp OR 'mandible' OR 'mandibular' OR 'jaws'/exp OR 'jaws' OR 'intraosseous' OR 'central'/exp OR 'central') | **1,555** |
| **TOTAL** |  | **3,325** |

**Table S2.** Excluded articles

| Author | Year | Country | Exclusion reason |
| --- | --- | --- | --- |
| Abbott | 2001 | Australia | Incomplete date |
| Ali et al | 2017 | USA | Not intraosseus ACC |
| Ali et al | 2016 | USA | Not intraosseus ACC |
| Bouaichi et al | 2014 | French | Incomplete date |
| Brookstone & Huevos | 1992 | USA | Incomplete date |
| Chang et al | 2018 | China | Not intraosseus ACC |
| Chen et al | 2014 | China | Incomplete follow-up |
| Closmann & Schmidt | 2006 | USA | Unavailable article |
| Collis et al | 2019 | Netherlands | Conference abstract |
| de Aquino et al | 2019 | Brazil | Incomplete follow-up |
| Deshpande et al | 2013 | India | Not intraosseus ACC |
| Erol et al | 2011 | Turkey | Unavailable article |
| Gamboa-Hoil et al | 2020 | Mexico | Not intraosseus ACC |
| Gingell et al | 1983 | USA | Incomplete follow-up |
| Gumgum et al | 2005 | Turkey | Incomplete follow-up |
| Hadfield et al | 1996 | UK | Not intraosseous ACC |
| He et al | 2016 | China | Language |
| Hersh et al | 2012 | USA | Conference abstract |
| Horré | 1974 | NA | Unavaliable article |
| Huang et al | 2021 | China | Not intraosseus ACC |
| Ishida et al | 2020 | Japan | Not intraosseus ACC |
| Juras et al | 2019 | Croatia | Not intraosseus ACC |
| Kalamchi et al | 1982 | NA | Not intraosseus ACC |
| Kimura et al | 2008 | Japan | Language |
| Kowalski & Paulino | 2002 | USA | Not intraosseus ACC |
| Lawal et al | 2017 | Nigeria | Incomplete follow-up |
| Lawrence, Johnson & Baker | 1971 | USA | Not intraosseous ACC |
| Li et al | 2015 | China | Language |
| Liu, Zhu & Chen | 2019 | China | Incomplete follow-up |
| Mathai et al | 2014 | India | Not intraosseus ACC |
| Oates, Kinsella & Gutowski | 2006 | UK | Not intraosseus ACC |
| Rayan & Wright | 2020 | Netherlands | Conference abstract |
| Ren et al | 2014 | China | Not intraosseus ACC |
| Santos et al | 2011 | Brazil | Incomplete date |
| Savithri et al | 2021 | India | Incomplete follow-up |
| Sengupta, Koley & Dutta | 1979 | India | Incomplete follow-up |
| Shamim et al | 2008 | India | Incomplete follow-up |
| Slavin & Mitchel | 1971 | UK | Incomplete follow-up |
| Tommasi & Leandro | 1972 | Brazil | Unavailable article |
| Uraizee, Cipriani & Ginat | 2008 | USA | Not intraosseus ACC |
| van Weert et al | 2011 | Netherlands | Conference abstract |
| Warren, Gnepp & Rosenblum | 1989 | USA | Unavailable article |
| Whitaker, Walshe & Hussain | 2014 | NA | Conference abstract |
| Yagihara et al | 2012 | Japan | Language |
| Yagihara et al | 2007 | Japan | Language |
| Yokokawa et al | 2021 | Japan | Not intraosseus ACC |
| Zaidi et al | 2016 | India | Conference abstract |

| Table S3. Included articles | | | | | | | | | | | | | | | | | | | | | |
| --- | --- | --- | --- | --- | --- | --- | --- | --- | --- | --- | --- | --- | --- | --- | --- | --- | --- | --- | --- | --- | --- |
| Author | **Year** | **Country** | **Studie type** | **N. of patients** | **Sex** | **Age** | **Affected** site | **Tumor site in the jaws** | **Symptoms** | **Clinical presentation** | **Evolution time (months)** | **Size (cm)** | **Palpable lymph node metastasis** | **Long Distance Metastasis** | **Radiographic findings** | **Histological grade** | **Perineural invasion** | **Primary treatment** | **Outcome** (mouths) | **Total follow-up (months)** | **Patient status during follow-up** |
| Al-Sukhun et al | 2006 | Finland | CR | 1 | F | 80 | Mandible | Parashynseal region | Pain | Intraoral swelling | NA | 3 | No | No | Irregular radiolucent | NA | NA | CS | No | 36 | NED |
| Brookstone, Huvos & Spiro | 1990 | USA | CR | 1 | F | 33 | Mandible | Body and ramus | Pain | Extraoral and intraoral swelling | 1 | NA | No | NA | NA | Cribriform | NA | RS + ND + RT | No | 16 | NED |
| Burkes et al | 1975 | USA | CR | 1 | F | 50 | Mandible | Parashynseal region | Pain | None | 16 | NA | NA | NA | Poor defined radiolucent area | NA | NA | RS | No | 36 | NED |
| Capodiferro et al | 2005 | Italy | CR | 1 | F | 36 | Mandible | Bilateral angle (2) | Paresthesia, pain | Extraoral and intraoral swelling | 2 | 3.9 x 3.6 | No | Yes (lungs) | Irregular radiolucent | NA | Yes | RS + RT + CT | No | 36 | AWD |
| Carlos-Bregni et al | 2009 | Brazil | CR | 1 | M | 46 | Mandible | Body and ramus | Paresthesia, pain | Intraoral swelling | NA | NA | No | Yes (lungs) | Poor defined radiolucent area | NA | NA | RS | No | 24 | NED |
| Clark, Triana & Meredith | 2000 | USA | CR | 1 | M | 54 | Mandible | Parashynseal region | None | Intraoral swelling | 3 | 10 x 8 | Yes | NA | NA | NA | NA | RS + ND + RT | No | 54 | NED |
| Cleveland et al | 1990 | USA | CS | 5 | M | 80 | Maxilla | Anterior | Swelling, Pain | Swelling | 6 | NA | NA | NA | Bone destruction, root resorption | Solid | No | RT | LDMet (lungs, bone), LNMet (NA) | 27 | DOD |
|  |  |  |  |  | M | 78 | Maxilla | Posterior and sinus | None | None | 12 | NA | NA | NA | Radiolucent area | Solid | No | RT | No | 84 | NED |
|  |  |  |  |  | F | 74 | Maxilla | Posterior and sinus | Paresthesia, pain, swelling, diplopia, headache | Swelling | Several | NA | NA | NA | Bone destruction | Solid | No | RT | LDMet (lungs, liver), local recurrence (13) | 15 | DOD |
|  |  |  |  |  | F | 59 | Maxilla | Posterior and sinus | Nonhealing extraction site | NA | NA | NA | NA | NA | Bone destruction | Solid | No | RT | NA | 22 | DOD |
|  |  |  |  |  | M | 39 | Maxilla | Posterior | Paresthesia | NA | NA | NA | NA | NA | Bone destruction | Solid | Yes | RS | Local recurrence (9) | 14 | DOD |
| Deng et al | 2014 | China | CS | 16 | F | 81 | Mandible | Body | Toothache | NA | NA | 5 x 3 | No | NA | NA | Solid | Yes | RS + ND | Met, local recurrence (29) | 29 | AWD |
|  |  |  |  |  | F | 46 | Mandible | Body | Swelling | NA | NA | 5 x 3 | Yes | NA | NA | Cribriform | NA | RS + RT | No | 76 | NED |
|  |  |  |  |  | F | 74 | Mandible | Body | Toothache | NA | NA | 6 x 6 | Yes | NA | NA | Solid | Yes | RS + ND + RT | No | 24 | DOD |
|  |  |  |  |  | M | 79 | Maxilla | NA | Paresthesia | NA | NA | 5 x 4 | No | NA | NA | Cribriform | NA | RS | No | 36 | NED |
|  |  |  |  |  | M | 30 | Maxilla | NA | Toothache | NA | NA | 4 x 3 | No | NA | NA | Cribriform | NA | RT + CT | No | 26 | NED |
|  |  |  |  |  | M | 47 | Maxilla | NA | Pain | NA | NA | 3 x 2 | No | NA | NA | Solid | NA | CS + RT | No | 25 | NED |
|  |  |  |  |  | M | 66 | Mandible | Ramus | Swelling | NA | NA | 5 x 4 | No | NA | NA | Tubular | NA | RS + RT | No | 21 | NED |
|  |  |  |  |  | F | 24 | Maxilla | NA | Swelling | NA | NA | 4 x 3 | Yes | NA | NA | Cribriform | NA | CS + RT | No | 180 | NED |
|  |  |  |  |  | F | 57 | Maxilla | NA | Swelling | NA | NA | 4 x 4 | No | NA | NA | Solid | Yes | CS + RT | Local recurrence (120) | 120 | AWD |
|  |  |  |  |  | M | 82 | Maxilla | NA | Swelling | NA | NA | 5 x 4 | No | NA | NA | Solid | NA | CS + RT | No | 24 | DOD |
|  |  |  |  |  | F | 63 | Mandible | NA | Swelling | NA | NA | 5 x 4 | No | NA | NA | Cribriform | Yes | RS + RT | Local recurrence (144) | 144 | AWD |
|  |  |  |  |  | F | 28 | Mandible | Body | Paresthesia | NA | NA | 5 x 3 | No | NA | NA | Tubular | NA | RS + ND + RT | No | 24 | NED |
|  |  |  |  |  | M | 66 | Maxilla | NA | Epistaxis | NA | NA | 3 x 2 | No | NA | NA | Solid | NA | RS + RT | No | 50 | NED |
|  |  |  |  |  | M | 65 | Mandible | Body | Swelling | NA | NA | 8 x 6 | Yes | NA | NA | Cribriform | NA | RS + RT | No | 74 | NED |
|  |  |  |  |  | F | 59 | Maxilla | NA | Swelling | NA | NA | 3 x 2 | No | NA | NA | Cribriform | NA | RS + RT | No | 39 | NED |
|  |  |  |  |  | F | 42 | Mandible | Anterior | Toothache, teeth mobility | NA | NA | 3 x 2 | No | NA | Poor defined radiolucent area | Cribriform | NA | RS + RT | No | 6 | NED |
| Elbeshir et al | 2014 | Sudan | CR | 1 | F | 47 | Mandible | Symphysis and parashynseal | Paresthesia, pain | Intraoral swelling | 4 | NA | No | No | Well-demarcad radiolucent area | Cribriform | NA | CS | No | 6 | NED |
| Favia et al | 2000 | UK | CR | 1 | F | 46 | Mandible | Anterior | Pain | Intraoral swelling | NA | 1.5 | No | No | Mixed lesion | Tubular/cribriform | No | RS | No | 168 | NED |
| Grimm et al | 2012 | Germany | CR | 1 | F | 45 | Mandible | Bilateral body and symphyseal region (2) | Paresthesia | Intraoral swelling | 18 | NA | No | No | Poor defined radiolucent area | NA | NA | RS + ND + RT | LDMet (lungs, breast, pancreas, kidney) (36) | 84 | DOD |
| Han et al | 2017 | China | CS | 4 | F | 57 | Mandible | Body and angle | Paresthesia, swelling | NA | 12 | 5 x 3 | NA | NA | Poor defined radiolucent area | Cribriform | No | RS + RT + CT | LDmet (lungs) (36) | 36 | AWD |
|  |  |  |  |  | M | 41 | Mandible | Body and ramus | Toothache, paresthesia, swelling | NA | 2 | 5 x 4 | Yes | NA | Poor defined radiolucent area | Solid | Yes | No treatment | LDMet (bone), LNMet (12) | 12 | AWD |
|  |  |  |  |  | M | 58 | Mandible | Body | Paresthesia, pain | Intraoral swelling | 2 | 3 x 2 | Yes | NA | Poor defined radiolucent area | Solid | Yes | RS + RT | No | 5 | NED |
|  |  |  |  |  | M | 54 | Mandible | Body | Swelling | NA | 24 | NA | NA | NA | Poor defined radiolucent area, root reabsorption | Cribriform | Yes | RS + ND + RT | No | 3 | NED |
| Hirota & Osaki | 1989 | Japan | CR | 1 | M | 82 | Mandible | Parashynseal and body | Swelling, pain | Extraoral and intraoral swelling | 12 | NA | No | NA | Poor defined radiolucent area | Cribriform | NA | RT + CT | No | 2 | DOC |
| Hu et al | 2017 | China | CS | 2 | F | 38 | Mandible | Posterior, symphysis (2) | Pain | Intraoral swelling | 12 | 2 | Yes | No | Poor defined radiolucent area | Cribriform | Yes | RS + ND + RT | No | 24 | NED |
|  |  |  |  |  | M | 52 | Mandible | Condyle | Pain | Intraoral swelling | 12 | 3 x 3 | No | Yes (lungs) | Poor defined radiolucent area | Cribriform | NA | CT | No | 24 | AWD |
| Indu & Roy | 2020 | India | CR | 1 | F | 71 | Mandible | Symphysis | Pain | Intraoral swelling | NA | 6 x 3 | No | No | Poor defined radiolucent area | Cribriform | No | S + RT | No | 15 | NED |
| Johnson, Millar & Leopard | 1989 | UK | CR | 1 | F | 68 | Mandible | Posterior | Swelling | Intraoral swelling | Several | 1 | No | No | Radiolucent area | Cribriform | NA | RS | Local recurrence (14) | 60 | AWD |
| Kaneda et al | 1982 | Japan | CR | 1 | M | 47 | Mandible | Posterior | Swelling | Extraoral and intraoral swelling | 1 | NA | Yes | No | Well-demarcad radiolucent area | Tubular/cribriform | NA | RS + ND | No | 60 | NED |
| Li et al | 2008 | China | CS | 3 | F | 38 | Maxilla | NA | Pain, loosing teeth | NA | NA | NA | NA | NA | NA | NA | NA | CS | Local recorrence (18) | 18 | AWD |
|  |  |  |  |  | F | 49 | Maxilla | NA | Pain, toothache, loosing teeth | NA | NA | NA | NA | NA | NA | NA | NA | RS + CT | No | 24 | NED |
|  |  |  |  |  | M | 47 | Maxilla | NA | Swelling | NA | NA | NA | NA | NA | NA | NA | NA | RS + CT | No | 12 | NED |
| Mano et al | 2010 | Japan | CR | 1 | M | 68 | Mandible | Symphysis | Swelling, paresthesia, losing teeth | Intraoral swelling | NA | NA | No | NA | Poor defined radiolucent area, root reabsorption | Cribriform | Yes | RS + RT + CT | LDMet (bone) (1) | 13 | DOD |
| de Marcos et al | 2007 | Spain | CR | 1 | F | 57 | Mandible | Body | Paresthesia | Teeth mobility | 2 | 3 | NA | NA | Poor defined radiolucent area | Tubular/cribriform | Yes | RS + RT | No | 36 | NED |
| Martinez-Madrigal et | 2000 | Mexico | CS | 4 | M | 51 | Mandible | Angle | NA | NA | NA | NA | NA | NA | NA | NA | NA | RS + RT | NA | 48 | DOD |
|  |  |  |  |  | M | 48 | Mandible | Body | NA | NA | NA | NA | NA | NA | NA | NA | NA | RS + RT | NA | 36 | DOD |
|  |  |  |  |  | F | 24 | Mandible | Angle | NA | NA | NA | NA | NA | NA | NA | NA | NA | RS + RT | NA | 84 | AWD |
|  |  |  |  |  | F | 35 | Mandible | Angle | NA | NA | NA | NA | NA | NA | NA | NA | NA | RS + RT | NA | 60 | AWD |
| Mahomed et al | 2009 | South Africa | CR | 1 | F | 53 | Mandible | Symphysis and body | Swelling | Extraoral swelling | 48 | NA | NA | NA | Multilocular mixed area | Tubular/cribriform | Yes | RS + RT | No | 36 | NED |
| Reddy et al | 2020 | India | CR | 1 | M | 55 | Mandible | Parashynseal and body | Swelling | Intraoral swelling | 12 | NA | No | No | Poor defined radiolucent area | Solid | NA | RS + ND | NA | 36 | DOD |
| Tseng et al | 2022 | China | CS | 1 | M | 56 | Mandible | Body | Pain | NA | NA | NA | NA | NA | Well-demarcad radiolucent area | NA | NA | CS | No | 192 | NED |
| Vinuth et al | 2013 | India | CR | 1 | M | 56 | Mandible | Symphysis and body | Pain | NA | 5 | NA | Yes | NA | Poor defined radiolucent area | Solid | Yes | RS + ND + RT | No | 15 | NED |
| Yoshimura et al | 1978 | Japan | CR | 1 | F | 47 | Mandible | Parashynseal | Paresthesia | None | 36 | NA | No | Yes (trigeminal ganglion) | Well-demarcad radiolucent area | Solid/cribriform | NA | RT | No | 34 | NED |
| Waechter et al | 2017 | Brazil | CR | 1 | F | 55 | Maxilla | Anterior | None | None | 12 | 1.5 | NA | NA | Well-demarcad radiolucent area | Tubular/cribriform | NA | RS + RT | No | 18 | NED |

*Legend:* AWD: alive with disease; CR: case report; CS: case serie; CS: conservatory surgery; DOC: dead of other cause; DOD: dead of disease; LDmet: long distance metastasis; LNmet: lymph node metastasis; NA: Not available; ND: neck dissection; NED: no evidence of disease; QT: chemotherapy; RS: radical surgery; RT: radiation therapy

**Figure S1.** Risk of bias (Joanna Briggs Institute)

*Case reports*


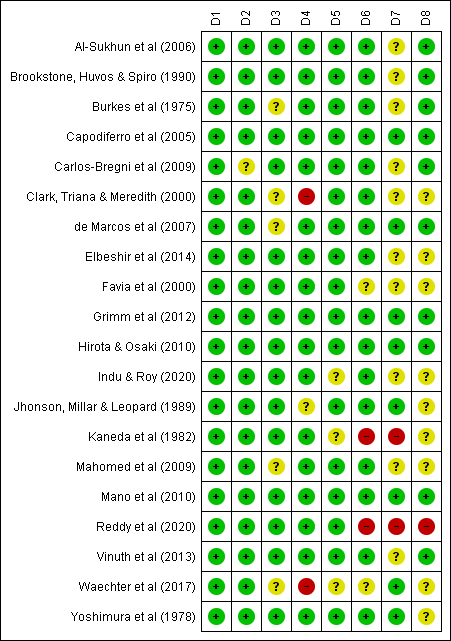


*Legend*: D1. Were patient’s demographic characteristics clearly described? D2. Was the patient’s history clearly described and presented as a timeline? D3. Was the current clinical condition of the patient on presentation clearly described? D4. Were diagnostic tests or assessment methods and the results clearly described? D5. Was the intervention(s) or treatment procedure(s) clearly described? D6. Was the post-intervention clinical condition clearly described? D7. Were adverse events (harms) or unanticipated events identified and described? D8. Does the case report provide takeaway lessons?

*Case serie*


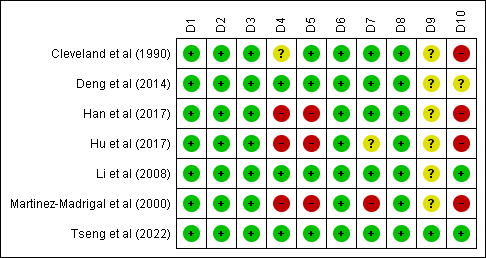


*Legend:* D1. Were there clear criteria for inclusion in the case series? D2. Was the condition measured in a standard, reliable way for all participants included in the case series? D3. Were valid methods used for identification of the condition for all participants included in the case series? D4. Did the case series have consecutive inclusion of participants? D5. Did the case series have complete inclusion of participants? D6. Was there clear reporting of the demographics of the participants in the study? D7. Was there clear reporting of clinical information of the participants? D8. Were the outcomes or follow up results of cases clearly reported? D9. Was there clear reporting of the presenting site(s)/clinic(s) demographic information? D10. Was statistical analysis appropriate?
